# Mitochondrial morphological aberrations and redistribution in podocytes with glomerular diseases

**DOI:** 10.1080/0886022X.2025.2515528

**Published:** 2025-07-09

**Authors:** Yu Wang, Xinying Yu, Zihan Wang, Shuang Yao, Huimin Ma, Zongda Li, Rui Zhang, Haihai Liang, Jundong Jiao

**Affiliations:** ^a^ Department of Nephrology, The Second Affiliated Hospital of Harbin Medical University, Harbin, China; ^b^ College of Pharmacy, Harbin Medical University, Harbin, China

**Keywords:** Glomerular disease, podocyte mitochondria, transmission electron microscopy, IgA nephropathy, minimal change nephropathy, idiopathic membranous nephropathy

## Abstract

**Introduction:**

The primary aim of this study was to investigate the morphological characteristics of podocyte mitochondria in glomerular diseases, and to analyze the correlations between these characteristics and podocyte injury, as well as clinical indicators.

**Methods:**

This study analyzed renal biopsies from 103 patients with IgA nephropathy (IgAN), minimal change nephropathy (MCN), and idiopathic membranous nephropathy (IMN), alongside 22 controls, using transmission electron microscopy (TEM). Mitochondrial size, shape, density, and subcellular distribution were quantified and correlated with podocyte injury (foot process width, FPW) and clinical parameters.

**Results:**

The results indicated that podocyte mitochondria in patients with glomerular diseases were smaller, rounder, and significantly more numerous, with a more pronounced increase in the region proximal to the glomerular basement membrane (GBM). The FPW indicators revealed that podocyte damage was most severe in the IMN group, followed by the MCN group, and least severe in the IgAN group. Mitochondrial size was negatively correlated with FPW, while mitochondrial density distribution was positively correlated with FPW. In the IgAN group, the morphological characteristics of podocyte mitochondria were statistically correlated with clinical parameters, including age, estimated glomerular filtration rate (eGFR), albumin levels, and proteinuria.

**Conclusion:**

Podocytes in patients with glomerular diseases exhibit significant mitochondrial morphological abnormalities, including mitochondrial fragmentation, increased fission, and altered distribution. Furthermore, in IgAN, mitochondrial morphological abnormalities and their redistribution in podocytes are significantly correlated with podocyte injury—primarily manifested as foot process effacement—and with the severity of the disease.

## Introduction

Glomerular disease poses a significant health challenge for millions of people worldwide, characterized by inflammation and structural damage that progressively lead to kidney function decline [1]. Podocytes, a key cellular component of the glomerular filtration barrier, exhibit pathological alterations that are a common feature of nearly all glomerular diseases [2,3]. The clinicopathological manifestations of podocyte injury primarily include foot process effacement [4], which are closely related to proteinuria and disease progression [5].

In recent years, research on podocyte mitochondria has garnered increasing attention [6,7]. Changes in mitochondrial morphology and quantity often reflect local metabolic demands and mitochondria play a crucial role in maintaining cellular homeostasis [8,9]. In glomerular diseases, mitochondrial morphological and functional abnormalities are considered key factors contributing to podocyte injury [7,10]. Studies on focal segmental glomerulosclerosis (FSGS) [11,12], diabetic nephropathy (DN) [13–15], and lupus nephritis (LN) [16,17] have identified mitochondrial abnormalities such as swelling, loss of cristae structure, and excessive fission as being associated with podocyte injury [13,17,18]. However, observations of podocyte mitochondria in human renal tissue across a broad spectrum of glomerular diseases remain limited, particularly regarding the subcellular distribution of mitochondria [19]. Therefore, it is necessary to investigate the correlation between podocyte mitochondrial damage and disease severity across a wider range of glomerular diseases.

Transmission electron microscopy (TEM) is an indispensable tool for investigating ultrastructural changes, particularly in mitochondria [20]. It provides high-resolution images that enable detailed visualization of mitochondrial size, shape, density, and distribution within podocytes [17,21,22]. In renal pathology, TEM has been extensively utilized to examine ultrastructural alterations in various kidney diseases [23], including glomerular disorders [24]. TEM offers a unique ability to scrutinize the fine details of podocyte mitochondria [25–27], which are often overlooked in clinical studies. This study employed TEM to observe podocyte mitochondria in common glomerular diseases such as IgA nephropathy (IgAN), minimal change nephropathy (MCN), and idiopathic membranous nephropathy (IMN), with the aim of addressing these frequently overlooked aspects.

## Materials and methods

### Subjects

A total of 103 patients (mean age 45.5 ± 14.2 years) with biopsy-proven glomerular diseases were recruited from the Department of Nephrology at the Second Affiliated Hospital of Harbin Medical University between 2023 and 2024. Glomerular disease diagnoses were established based on renal biopsy findings. The cohort included 39 cases of IgAN, characterized by predominant IgA deposition within the mesangial region of the glomeruli as confirmed by immunofluorescence; 20 cases of MCN, in which neither immunoglobulin nor complement deposition was detected by immunofluorescence, accompanied by diffuse podocyte foot process effacement observed on electron microscopy; and 44 cases of IMN, identified by the presence of subepithelial IgG deposits along the GBM. Patients with glomerular diseases secondary to systemic conditions such as lupus, hepatitis B virus infection, IgA vasculitis, diabetes mellitus, or renal hypertension were excluded from the study. As normal controls, renal tissue adjacent to tumors from 22 patients who underwent nephrectomy for renal tumors was utilized. The control group had no history of hypertension, diabetes, or primary glomerular diseases, and the age of the control subjects (46.8 ± 10.8 years) was comparable to that of the glomerular disease group.

### Clinical data and laboratory tests

Clinical data and laboratory test results at the time of renal biopsy were collected, including age, sex, systolic and diastolic blood pressure, uric acid, serum creatinine, urea, serum albumin, and proteinuria. The estimated glomerular filtration rate (eGFR) was calculated using the CKD-EPI equation (Chronic Kidney Disease Epidemiology Collaboration) [28].

### Transmission electron microscopy specimen preparation and image acquisition

Renal biopsy specimens for TEM analysis were fixed in 2.5% glutaraldehyde phosphate buffer, post-fixed in 1% osmium tetroxide, and embedded in Epon 812 resin. Ultrathin sections were stained with uranyl acetate and lead citrate and examined using a transmission electron microscope (HT7700 Exalens, Japan). Under the TEM, glomeruli were identified, and the magnification was set to 20,000×. Starting from a point at the edge of the glomerulus, a zigzag scanning pattern was employed. Images were captured whenever podocytes appeared in the field of view, continuing until the entire glomerulus was scanned [17]. We recorded the number of podocytes in each glomerulus and selected podocyte images from different fields of view for each sample for analysis.

### Mitochondria morphometry

Mitochondria in podocytes were measured under TEM using Image-Pro Plus software [29]. For each patient, at least 50 mitochondria were analyzed. The measurement data included length, perimeter, area, aspect ratio (major axis length/minor axis length), circularity [4π(Area/Perimeter^2^)], and roundness [4 * Area/(π * Lengt h^2^)] [30–32]. The average of these indicators across all mitochondria for each patient was considered the final result for that indicator.

### Mitochondrial density and distribution

**Figure 1.**
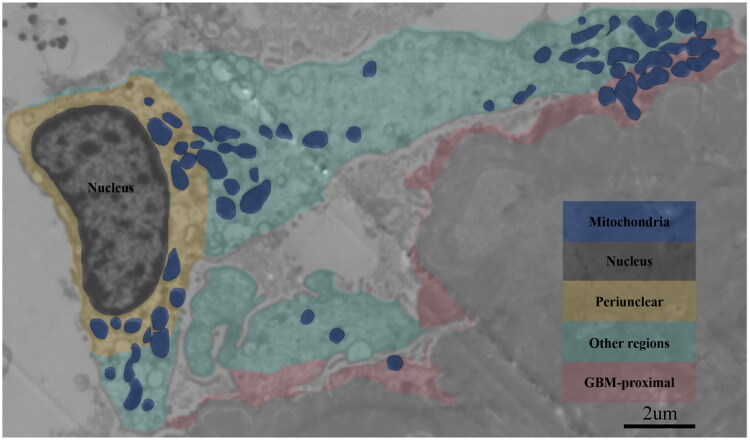
Measurement analysis scheme for mitochondrial size, shape, density, and distribution under electron microscopy.

In this study, mitochondrial density in podocytes was quantified using numerical density (number of mitochondria per unit cytoplasmic area) [17,33]. To assess mitochondrial distribution, their intracellular locations were categorized into three regions: perinuclear (within 1 μm of the nucleus), proximal to the GBM (within 1 μm of the GBM), and other regions (excluding perinuclear and GBM-proximal region) [34]. The numerical density of mitochondria was calculated for each of these regions (Figure 1).

### Foot process width (FPW)

The length of the GBM in each TEM image was measured using Image-Pro Plus. The number of slit diaphragms along the GBM was manually counted. Foot processes were defined as contiguous epithelial segments between two adjacent slit diaphragms and in contact with the GBM. The foot process width was calculated as follows: FPW = (π/4) × (ΣGBM length/Σfoot processes), where ΣGBM length is the total length of the GBM measured in each image, and Σfoot processes is the total number of foot processes counted on the peripheral GBM. The factor π/4 is applied to correct for random variations that may occur due to the angle of intersection relative to the long axis of the foot processes [35,36]. The average FPW value for each patient was used as the final result for this index.

### Statistical analysis

Continuous variables with a normal distribution were expressed as mean ± standard deviation (mean ± SD), while those with a non-normal distribution were presented as median and interquartile range (median [IQR]). One-way analysis of variance (ANOVA) was used to compare normally distributed variables, and the Kruskal-Wallis H test was applied for non-normally distributed variables. Spearman’s correlation coefficient (*r*) was used to evaluate associations between continuous clinical and pathological parameters. Statistical analyses were performed using SPSS 26.0 software (IBM, Armonk, NY, USA) and GraphPad Prism 10. All *p*-values were two-sided, and differences were considered statistically significant if *p* < 0.05.

## Results

### Clinical characteristics and laboratory findings of patients

A total of 125 renal biopsy samples were included in this study, comprising 22 samples from peritumoral tissues of renal tumor patients, 39 from patients with IgAN, 20 from patients with MCN, and 44 from patients with IMN. The average age of the patients was 45.6 ± 13.7 years, with 79 male patients. Blood pressure measurements showed no significant differences between groups.

Significant differences in proteinuria were observed among the IMN, MCN, and IgAN groups (*p* < 0.05). Serum albumin levels differed significantly compared to the control group (*p* < 0.05) and also varied significantly between the IgAN group and the other groups (*p* < 0.05), while no significant difference was observed between the IMN and MCN groups. No statistically significant differences were found among the groups for serum creatinine, eGFR, or uric acid levels (Table 1).

**Table 1.** The clinical characteristics of study subjects.

	Control (*n* = 22)	IgAN (*n* = 39)	MCN (*n* = 20)	IMN (*n* = 44)
Age (years)	46.8 ± 10.8	40.1 ± 14.2	41.9 ± 17.3	51.9 ± 9.5^b,c^
Sex (male/female)	15/7	24/15	14/6	26/18
Systolic BP (mmHg)	125.1 ± 8.3	127.6 ± 16.2	123.4 ± 13.0	131.0 ± 10.2
Diastolic BP (mmHg)	80.0 ± 4.7	82.7 ± 9.6	82.1 ± 6.6	82.4 ± 6.1
Uric acid (µmol/L)	325.3 ± 81.4	329.2 ± 84.7	389.9 ± 83.2	342.1 ± 84.8
Serum creatinine (μmol/L)	73.0 ± 15.5	88.7 ± 26.4	90.2 ± 31.4	75.5 ± 19.4
eGFR (mL/min/1.73 m^2^)	102.1 ± 16.8	83.0 ± 29.3	89.1 ± 32.4	94.9 ± 18.6
Urea (mmol/L)	5.5 ± 1.3	6.1 ± 2.2	7.0 ± 3.3	5.9 ± 1.3
Albumin (g/L)	44.4 ± 3.7	37.1 ± 7.6^a^	20.3 ± 8.3^a,b^	24.9 ± 6.1^a,b^
Proteinuria (g/day)	NA	1.4 (0.6, 3.3)	9.0 (4.3, 14.0)^b^	9.5 (6.5, 13.1)^b^

a: vs Control, *p* < 0.05. b: vs IgAN, *p* < 0.05. c: vsMCN, *p* < 0.05.

### Mitochondrial morphological abnormalities and distribution changes in podocytes of patients with glomerular diseases

**Figure 2.**
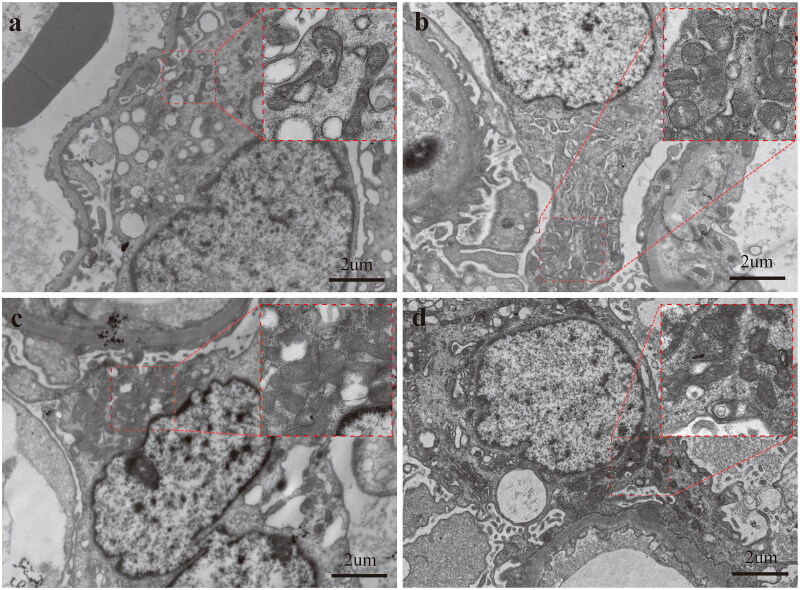
Mitochondrial morphological aberrations and redistribution in podocytes across various glomerular diseases. (a) In the control group, podocyte mitochondria exhibit elongated morphology. In the IgAN group (b), MCN group (c) and IMN group (d), podocyte mitochondria display altered morphology and redistribution. Magnification: 20,000x. Scale bar: 2 µm.

Through electron microscopy examination of renal biopsy samples from patients, we observed significant mitochondrial fission and a variety of abnormal mitochondrial structures in some podocytes, including mitochondrial swelling, loss of cristae, and membrane rupture. These abnormalities were commonly observed in patients with IgAN, MCN, and IMN (Figure 2). We quantified mitochondrial morphological abnormalities by measuring mitochondrial size, shape, density, and distribution within podocytes [17,31].

### Mitochondrial size

The size of podocyte mitochondria was statistically analyzed using electron microscopy. The results demonstrated that mitochondrial length in the IgA group (0.473 ± 0.060 µm, *p* < 0.01), MCN group (0.472 ± 0.073 µm, *p* < 0.001), and IMN group (0.446 ± 0.045, *p* < 0.0001) was significantly shorter compared to the control group (0.551 ± 0.076 µm). Additionally, the mitochondrial perimeter in the IgA group (1.266 ± 0.145 µm, *p* < 0.05), MCN group (1.272 ± 0.190 µm, *p* < 0.01), and IMN group (1.204 ± 0.126 µm, *p* < 0.0001) was significantly smaller than in the control group (1.434 ± 0.196 µm). In terms of mitochondrial area, the IgA group (0.103 ± 0.023 µm^2^), MCN group (0.106 ± 0.031 µm^2^), and IMN group (0.088 ± 0.017 µm^2^, *p* < 0.0001) exhibited significantly smaller areas compared to the control group (0.121 ± 0.034 µm^2^). Notably, the IMN group showed particularly significant differences relative to the control group. These findings suggest that during podocyte injury, mitochondria become more fragmented [37], a phenomenon especially pronounced in the IMN group (Table 2).

**Table 2.** Mitochondrial size, shape, density, and distribution of study subjects.

	Control	IgAN	MCN	IMN
Mitochondrial size				
Length (µm)	0.551 ± 0.076	0.473 ± 0.060^a^	0.472 ± 0.073^a^	0.446 ± 0.045^a^
Perimeter (µm)	1.434 ± 0.196	1.266 ± 0.145^a^	1.272 ± 0.190^a^	1.204 ± 0.126^a^
Area (µm²)	0.121 ± 0.034	0.103 ± 0.023	0.106 ± 0.031	0.088 ± 0.017^a,b^
Mitochondrial shape				
Aspect ratio	2.189 ± 0.358	1.861 ± 0.278^a^	1.836 ± 0.266^a^	1.915 ± 0.229^a^
Circularity	0.738 ± 0.078	0.792 ± 0.054^a^	0.792 ± 0.063^a^	0.765 ± 0.047
Roundness	0.546 ± 0.081	0.624 ± 0.065^a^	0.634 ± 0.063^a^	0.609 ± 0.061^a^
Mitochondrial density				
Total	0.277 ± 0.120	0.328 ± 0.098	0.402 ± 0.072^a,b^	0.447 ± 0.056^a,b^
Periunclear	0.309 ± 0.155	0.436 ± 0.155^a^	0.466 ± 0.130^a^	0.470 ± 0.102^a^
Other regions	0.339 ± 0.137	0.396 ± 0.116	0.406 ± 0.097	0.514 ± 0.085^a,b,c^
GBM-proximal	0.065 ± 0.054	0.158 ± 0.101^a^	0.209 ± 0.086^a^	0.255 ± 0.123^a^

a: vs Control, *p* < 0.05. b: vs IgAN, *p* < 0.05. c: vsMCN, *p* < 0.05.

### Mitochondrial shape

The shape of podocyte mitochondria was statistically analyzed using electron microscopy. The results demonstrated that the aspect ratio of mitochondria in the IgA group (1.861 ± 0.278, *p* < 0.05), MCN group (1.836 ± 0.266, *p* < 0.01), and IMN group (1.915 ± 0.229, *p* < 0.05) was significantly lower compared to the control group (2.189 ± 0.358), indicating a change in mitochondrial morphology [15]. In terms of circularity, the IgA group (0.792 ± 0.054, *p* < 0.01), MCN group (0.792 ± 0.063, *p* < 0.05), and IMN group (0.765 ± 0.047) exhibited higher values than the control group (0.738 ± 0.078). Regarding roundness, the IgA group (0.624 ± 0.065, *p* < 0.001), MCN group (0.634 ± 0.063, *p* < 0.001), and IMN group (0.609 ± 0.061, *p* < 0.01) also showed higher values compared to the control group (0.546 ± 0.081). The increased circularity and roundness values in the disease groups suggest that mitochondria became more spherical. This may be related to mitochondrial swelling or fission [31,38] (Table 2).

### Mitochondrial density and distribution

**Figure 3.**
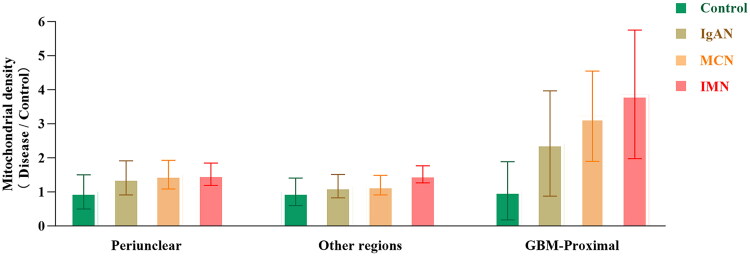
Increasing trend of mitochondrial density in different subcellular regions. The figure illustrates the relative increase in mitochondrial density across various subcellular regions in each disease group compared to the control group. In the perinuclear region, mitochondrial density progressively increased in all disease groups, with the IMN group showing the highest fold increase (1.521-fold). Similarly, mitochondrial density increased in other cellular regions, with the IMN group exhibiting the most significant increase (1.516-fold). Notably, in the region proximal to the GBM, mitochondrial density showed a significant increase, with the IMN group demonstrating the highest fold increase (3.864-fold), followed by the MCN group (3.221-fold) and the IgAN group (2.423-fold).

The density and distribution of podocyte mitochondria were statistically analyzed using electron microscopy. The results demonstrated that mitochondrial density was significantly higher in the IMN group (0.447 ± 0.056, *p* < 0.0001) and the MCN group (0.402 ± 0.072, *p* < 0.001) compared to the control group (0.277 ± 0.120). Although mitochondrial density in the IgAN group (0.328 ± 0.098) also increased, this difference was not statistically significant (Table 2). Overall, our observations of smaller, rounder, and more densely packed mitochondria suggest that mitochondrial fission is enhanced in disease states [39–41]. Furthermore, in podocytes from patients with glomerular diseases, the density of mitochondria in the perinuclear region, GBM-proximal region, and other cellular regions was significantly elevated, consistent with the overall increase within the cell [42]. Notably, this trend was particularly pronounced in the region proximal to the GBM (Figure 3), indicating that in glomerular diseases, podocyte mitochondria may be redistributed toward the GBM region [43].

### Podocyte injury in different glomerular diseases

Significant differences in podocyte injury were observed among various disease groups, as measured by FPW using electron microscopy images. The median FPW was highest in the IMN group at 2.713 (1.963, 4.319) µm, followed by the MCN group at 1.728 (1.204, 2.182) µm, and the IgAN group at 0.785 (0.497, 1.571) µm. The control group exhibited the lowest median FPW at 0.576 (0.491, 0.645) µm. Statistically significant increases in FPW were observed in all disease groups compared to the control group (*p* < 0.05) [44]. However, the podocyte number evaluated by TEM did not show statistically significant differences among the groups (Table 3) [45].

**Table 3.** The degree of podocyte injury between different disease.

	Control (*n* = 22)	IgAN (*n* = 39)	MCN (*n* = 20)	IMN (*n* = 44)
Foot process width (µm)	0.576^b,c,d^ (0.491, 0.645)	0.785^a,c,d^ (0.497, 1.571)	1.728^a,c,d^ (1.204, 2.182)	2.713^a,b,c^ (1.963, 4.319)
Number of evaluated podocytes in TEM	20.67 ± 5.993	19.66 ± 5.401	19.18 ± 6.354	18.00 ± 5.112

a: vs Control, *p* < 0.05. b: vs IgAN, *p* < 0.05. c: vsMCN, *p* < 0.05. d: vs IMN, *p* < 0.05.

### Correlation between podocyte mitochondrial morphology and podocyte injury

We analyzed the relationship between podocyte injury and mitochondrial morphology. The results showed that mitochondrial size parameters (length, perimeter, area) were significantly negatively correlated with podocyte injury (*p* < 0.01 in the total sample). In contrast, mitochondrial density parameters (total density, perinuclear density, other regions density, GBM-proximal density) were significantly positively correlated with podocyte injury (*p* < 0.01 in the total sample). However, when association analysis was performed across the glomerular disease groups, only in IgAN groups were mitochondrial size (*p* < 0.05) and increased density (*p* < 0.01) consistent with the overall trend. In IMN groups, only the density proximal to the GBM was significantly correlated with podocyte injury (*p* < 0.05). Mitochondrial shape parameters (aspect ratio, circularity, roundness) did not show statistically significant correlations in any of the three groups (Table 4). These findings suggest that as podocyte injury worsens, mitochondrial fragmentation increases and mitochondrial fission becomes more pronounced in IgAN [17].

**Table 4.** The correlation of podocyte damage with mitochondrial size, shape and density distribution.

	Total		IgAN		MCN		IMN	
	*r*	*p*	*r*	*p*	*r*	*p*	*r*	*p*
Mitochondrial size								
Length (µm)	−0.345	<0.01*	−0.374	<0.05*	−0.199	0.41	−0.178	0.25
Perimeter (µm)	−0.319	<0.01*	−0.398	<0.05*	−0.134	0.58	−0.166	0.29
Area (µm²)	−0.354	<0.01*	−0.402	<0.05*	−0.054	0.83	−0.129	0.41
Mitochondrial shape								
Aspect ratio	0.038	0.71	−0.061	0.71	−0.250	0.30	−0.058	0.71
Circularity	−0.169	0.09	0.073	0.66	0.050	0.84	−0.069	0.66
Roundness	−0.052	0.61	0.148	0.37	0.133	0.59	0.089	0.57
Mitochondrial density								
Total	0.589	<0.01*	0.478	<0.01*	0.041	0.87	0.343	0.05
Periunclear	0.346	<0.01*	0.503	<0.01*	0.223	0.36	0.142	0.44
Other regions	0.506	<0.01*	0.288	0.08	0.168	0.49	0.164	0.36
GBM-proximal	0.594	<0.01*	0.769	<0.01*	0.051	0.84	0.428	<0.05*

Data were analyzed by Spearman. *r*: Spearman’s correlation coefficient. *p* < 0.05 was set as the significance threshold, *Significant values.

### Correlation between podocyte mitochondrial morphology and laboratory parameters

We analyzed the correlation between mitochondrial morphological features and laboratory parameters across different disease groups. Significant correlations were observed only in the IgAN group, with no significant correlations found in the MCN or IMN groups. In the IgAN group, mitochondrial shape parameters (circularity, roundness) were positively correlated with age (*p* < 0.05), indicating that podocyte mitochondria became more spherical as age increased. Additionally, mitochondrial size parameters (length, perimeter, area) were negatively correlated with proteinuria (*p* < 0.01) and positively correlated with serum albumin levels (*p* < 0.01). Mitochondrial density parameters (total density, perinuclear density, other regions density, GBM-proximal density) were positively correlated with proteinuria and negatively correlated with serum albumin levels. Notably, mitochondrial density proximal to the GBM was also negatively correlated with eGFR (*p* < 0.05) (Table 5). These results suggest that podocyte mitochondria aberrations are associated with the severity of IgAN.

**Table 5.** The correlation of laboratory data with mitochondrial size, shape, and density distribution in IgAN.

	Age		eGFR		Albumin		Proteinuria	
	*r*	*p*	*r*	*p*	*r*	*p*	*r*	*p*
Mitochondrial size								
Length (µm)	−0.153	0.36	0.101	0.56	0.557	<0.01*	−0.465	<0.01*
Perimeter (µm)	−0.066	0.69	0.082	0.63	0.502	<0.01*	−0.479	<0.01*
Area (µm²)	0.065	0.70	0.081	0.64	0.442	<0.01*	−0.445	<0.01*
Mitochondrial shape								
Aspect ratio	−0.301	0.07	0.014	0.93	0.241	0.14	−0.083	0.62
Circularity	0.331	<0.05*	0.066	0.69	−0.161	0.33	0.056	0.73
Roundness	0.333	<0.05*	−0.055	0.75	−0.281	0.08	0.144	0.38
Mitochondrial density								
Total	0.145	0.39	−0.140	0.42	−0.626	<0.01*	0.672	<0.01*
Periunclear	0.157	0.35	−0.197	0.25	−0.667	<0.01*	0.585	<0.01*
Other regions	0.031	0.85	0.003	0.99	−0.484	<0.01*	0.428	<0.01*
GBM-proximal	0.311	0.06	−0.333	<0.05*	−0.488	<0.01*	0.590	<0.01*

Data were analyzed by Spearman. *r*: Spearman’s correlation coefficient. *p* < 0.05 was set as the significance threshold, *Significant values.

## Discussion

In this study, we observed that despite the different pathogenic mechanisms of various glomerular diseases, podocyte mitochondria exhibited similar pathological morphological changes. Compared to the control group, podocyte mitochondria in the glomerular disease groups were generally smaller, more spherical, and more numerous, with a particularly pronounced increase in density near the GBM. These findings suggest that the ultrastructural pathological features of podocyte mitochondria are consistent across different glomerular diseases [15,17]. Moreover, we found that the severity of podocyte mitochondrial abnormalities in IgAN group could reflect the degree of podocyte injury. To quantify the degree of podocyte injury, we measured the FPW and podocyte number. The results demonstrated that, compared to the control group, FPW was significantly correlated with disease severity in IgAN, MCN, and IMN; however, the number of podocytes did not show statistical significance. Although overall mitochondrial morphological parameters were significantly correlated with FPW in the glomerular disease groups, subgroup analysis revealed that the correlation between podocyte mitochondrial morphology and FPW was only observed in the IgAN group. We speculate that this may be due to the heterogeneity of podocyte injury within the IgAN group, which includes cases with relatively mild podocyte injury [46]. Consequently, IgAN serves as an ideal model for observing the gradient of podocyte injury, allowing us to detect trends consistent with podocyte mitochondrial injury. In contrast, podocytes in patients with IMN and MCN exhibited more pronounced and homogeneous foot process effacement. This uniform pathological alteration suggests that podocytes in these patients are subjected to more severe injury compared to those in the non-nephrotic IgA nephropathy group. At the same time, this also renders FPW ineffective in assessing the degree of podocyte injury. Additionally, the finding that the correlation between podocyte mitochondrial morphological abnormalities and redistribution with clinical indicators was only observed in the IgA nephropathy group further implies that mitochondrial injury in podocytes may play an important role in the early stages of podocyte injury, whereas more advanced stages of podocyte injury may involve additional pathogenic mechanisms [47]. Of course, whether mitochondrial morphological alterations are a causative factor for podocyte injury or merely a consequence of podocyte damage remains to be further elucidated by future research.

Under physiological conditions, although the mitochondrial density in podocytes is relatively low, it is sufficient to meet the energy demands of podocyte activities [19,48]. This energy is primarily used to maintain the actin cytoskeleton and regulate extracellular matrix proteins [10]. In disease groups, podocyte mitochondria also exhibit more abnormal morphologies, including mitochondrial swelling, loss of cristae, and outer membrane rupture. These abnormalities are rarely observed in normal podocytes and typically indicate podocyte injury [17]. Persistent mitochondrial swelling can lead to the irreversible opening of the mitochondrial permeability transition pore (mPTP), thereby increasing osmotic pressure within the mitochondrial matrix [49]. This ultimately results in the rupture of the outer mitochondrial membrane, the release of cytochrome c, and the activation of apoptotic caspases, triggering apoptosis [50]. Moreover, in disease groups, some podocyte mitochondria lack complete cristae, which is consistent with the description of mitochondrial cristae remodeling in podocytes under pathological conditions in DN mouse models by Koki Mise et al. [13]. Recent studies have shown that mitochondria lacking cristae may represent a unique subpopulation that does not directly participate in ATP synthesis but is mainly involved in reductive biosynthetic pathways, particularly the synthesis of proline and ornithine [51]. This may be related to the production of extracellular matrix proteins such as collagen [52]. Furthermore, the maintenance of mitochondrial morphology is crucial for podocyte function [7], and its morphological changes directly reflect the state of mitochondrial dynamics, a dynamic balance regulated by fission proteins such as Drp1 and fusion proteins such as Mfn1, Mfn2, and OPA1 [53,54]. In IgAN, the expression of Mfn2 is negatively correlated with the degree of podocyte injury and renal function indicators [55]. In childhood membranous nephropathy (CMN), the expression of mitochondrial fission proteins such as Drp1 and Fis1 increased, although there was no significant correlation with podocyte mitochondrial density or urinary protein levels [56]. Disruption of mitochondrial dynamics not only leads to mitochondrial dysfunction but also causes excessive production of reactive oxygen species (ROS), calcium ion imbalance, and reduced ATP production, ultimately leading to cell injury and the development of pathological states [53]. Under high-glucose conditions, an increase in mitochondrial-associated endoplasmic reticulum membrane (MAM) in podocytes promotes mitochondrial fission through the AKAP1-Drp1 signaling pathway, leading to podocyte injury [31]. In diabetic nephropathy (DN), increased mitochondrial fission in podocytes is associated with higher proteinuria levels [15]. The abnormal morphology of podocyte mitochondria observed in this study, characterized by reduced size, increased roundness, and increased number, suggests a disruption of mitochondrial dynamics [17].

The distribution of mitochondria within a cell is often determined by various parameters, including cell shape, cytoskeleton organization, and energy demands [43]. Podocyte mitochondria are primarily concentrated in the cytoplasm and primary foot processes, while they are rarely observed in secondary foot processes [33]. This distribution pattern is consistent with our observations in the control group. The limited space in secondary foot processes is insufficient to accommodate mitochondria; therefore, glycolysis provides ATP in the cortical region, while mitochondrial respiration supplies ATP in the cytoplasm around the cell nucleus [57]. However, in disease states, the distribution pattern of podocyte mitochondria changes, particularly in cases of foot process effacement, with a significant increase in mitochondrial density proximal to the GBM. This suggests that mitochondria may be redistributed within podocytes. The cause of this redistribution phenomenon remains unclear, but we speculate that it may be due to two factors. First, as foot process effacement increases, secondary foot processes disappear and widen, providing ample space for mitochondria to accumulate near the GBM. Second, the disappearance of foot processes disrupts the original glycolytic functional system, necessitating mitochondrial redistribution to meet the cell’s energy needs [58], such as maintaining and reorganizing the actin cytoskeleton or synthesizing slit diaphragm proteins and GBM components [10]. Existing studies have shown that mitochondria can move and position themselves within the cell through interactions with the cytoskeleton, redistributing according to the cell’s metabolic needs [4]. For example, during the invasion of the GBM by Caenorhabditis elegans cells, mitochondria cluster proximal to the GBM region before invasion and colocalize with the actin network, providing ATP locally to promote GBM rupture [59]. This study only observed the phenomenon of mitochondrial redistribution in podocytes under injury conditions, and the specific mechanisms and significance require further research and discussion.

This study has several limitations that merit consideration. First, mitochondrial quantification was performed on renal biopsy specimens, which may not fully capture the overall mitochondrial status throughout the kidney or in other affected tissues of the patients. Second, the single-center, retrospective design inherently limits the generalizability of our findings, as regional or population-specific factors may influence the results. Third, the glomerular diseases analyzed in this study—IgAN, MCN, and IMN—represent the most common pathological types at our institution; FSGS was excluded due to the limited number of cases diagnosed at our center. Fourth, the use of FPW as the main indicator of podocyte injury may not reflect all aspects of cellular damage. Fifth, the observational design and relatively small sample size increase the potential for selection bias. Finally, although TEM provided valuable ultrastructural information, the absence of complementary molecular or functional analyses restricts the elucidation of underlying mechanisms. Future studies incorporating multi-omics approaches and longitudinal designs are needed to validate these findings and to further explore the relevant pathogenic pathways.

## Data Availability

The data underlying this article will be shared upon reasonable request by the corresponding authors.
